# Supplementary material for: Antimicrobial resistance and genetic relationships of enterococci from siblings and non-siblings Heliconius erato phyllis caterpillars
Source: PeerJ. 2020 Feb 27;8:e8647. doi: 10.7717/peerj.8647 (PMC7049460; doi:10.7717/peerj.8647)

**Raw Data**

**Figure 1:** Genus-specific PCR assay targeting the *tuf* gene. Lane 1, 2 and 3: DNA standard, positive control *E. faecalis* ATCC 29212 (112 bp) and negative control, respectively. Lanes 4 to 16: strains 18.4, 18.5, 18.6, 18.7, 18.9, 18.10, 18.11, 18.13, 18.14, 18.15, 18.16, 18.17, and 18.18.

**STD
bp**

**1500-**

**1 2 3 4 5 6 7 8 9 10 11 12 13 14 15 16**


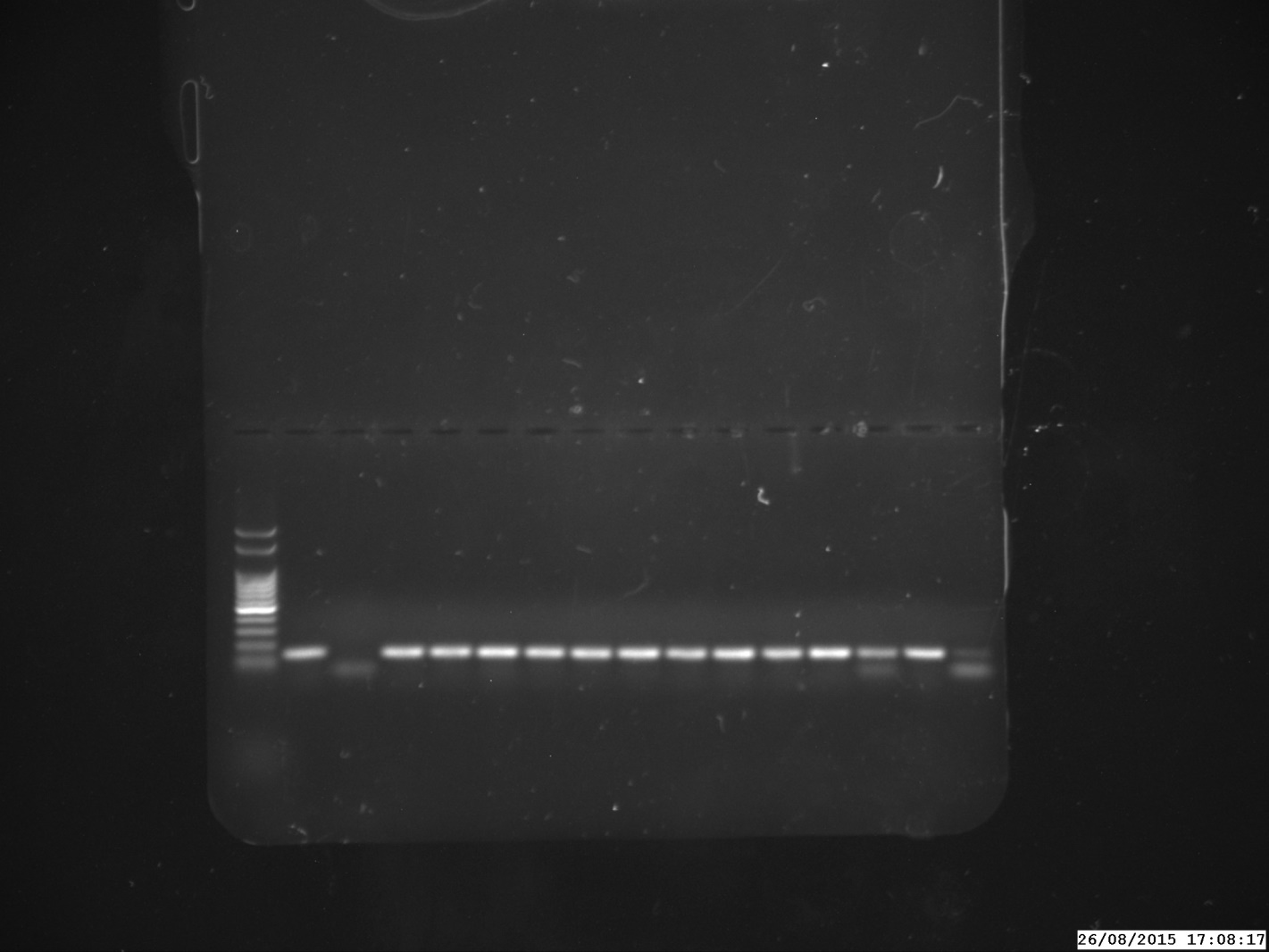


**1000-**

**500-**

**100-**

**M C+ C- 18.3 18.4 18.5 18.6 18.7 18.9 18.10 18.11 18.13 18.14 18.15 18.16 18.17**

**Figure 2:** Specie-specific PCR assay for identification of enterococci. **(A)** *E. casseliflavus*. Lanes 1, 2 and 3: DNA standard, positive control *E. casseliflavus* J6 (288 bp) and negative control, respectively. Lanes 4 to 16: strains 9.9, 9.10, 9.14, 9.15, 9.17, 9.18, 9.19, 9.20, 9.21, 9.22, 9.24, 18.1 and 18.2. **(B)** *E. mundtii*. Lanes 1 and 2: positive control *E. mundtii* J5 (94 bp) and negative control, respectively. Lanes 3 to 9: strains 29.14, 29.20, 26.19, 3.16, 3.18, 3.4. **(C)** *E. faecalis*. Lanes 1, 2 and 3: DNA standard, positive control *E. faecalis* ATCC 29212 (138 bp) and negative control, respectively. Lanes 4 to 16: strains 6.5, 6.18, 6.19, 10.6, 14.1, 14.2, 14.3, 14.4, 14.7, 14.8, 14.9, 14.10 and 14.17.

**1 2 3 4 5 6 7 8 9**

**STD
bp**

**1500-**

**1 2 3 4 5 6 7 8 9 10 11 12 13 14 15 16**


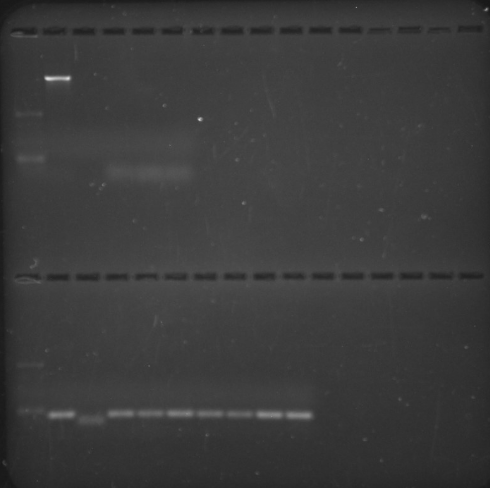




**94 bp-**

**(B)**

**(A)**

**500-**

**100-**

**1000-**

**STD
bp**

**1500-**

**1 2 3 4 5 6 7 8 9 10 11 12 13 14 15 16**





**(C)**

**1000-**

**500-**

**100-**

**Figure 3:** PCR assay for erythromycin-resistance encoding genes. (A) PCR assay for *erm*(B) gene. Lane 1, 2 and 3: DNA standard, positive control *Enterococcus* sp. 485 (639 bp) and negative control, respectively. Lanes 4 to 16: strains 11.9, 9.2, 9.5, 9.6, 9.7, 9.8, 9.9, 9.10, 9.14, 9.15, 9.17, 9.18 and 9.19. **(B)** PCR assay for *msr*C gene. Lane 1, 2 and 3: DNA standard, positive control *E. faecium* P4-2 (343 bp) and negative control, respectively. Lanes 4 to 16: strains 9.5, 9.6, 9.7, 9.8, 9.9, 9.10, 9.14, 9.15, 9.17, 9.18, 9.19, 9.20 and 9.21.

**STD
bp**

**1500-**

**STD
bp**

**1500-**

**1 2 3 4 5 6 7 8 9 10 11 12 13 14 15 16**

**1 2 3 4 5 6 7 8 9 10 11 12 13 14 15 16**

**
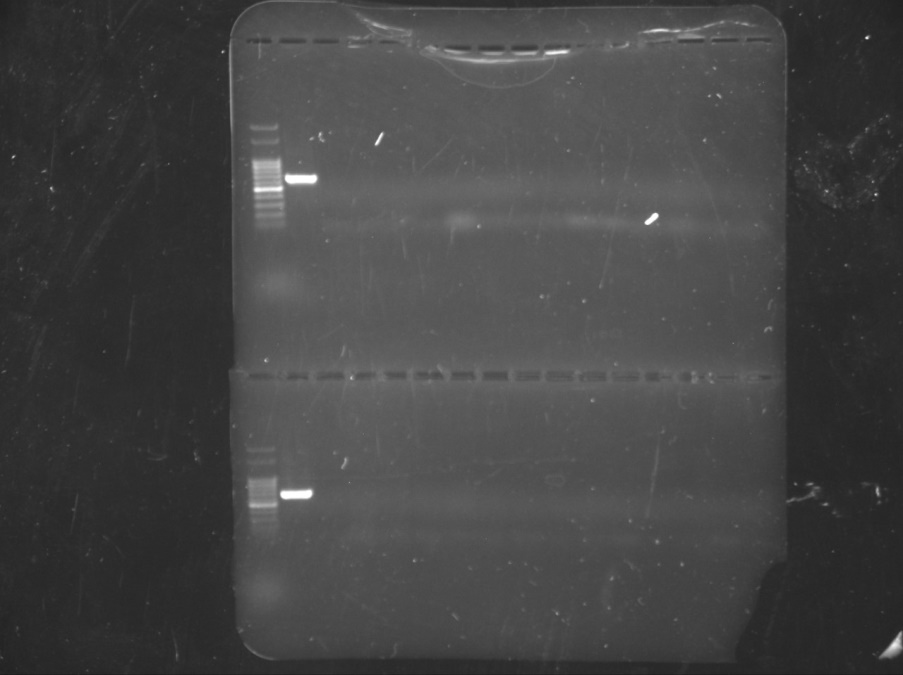

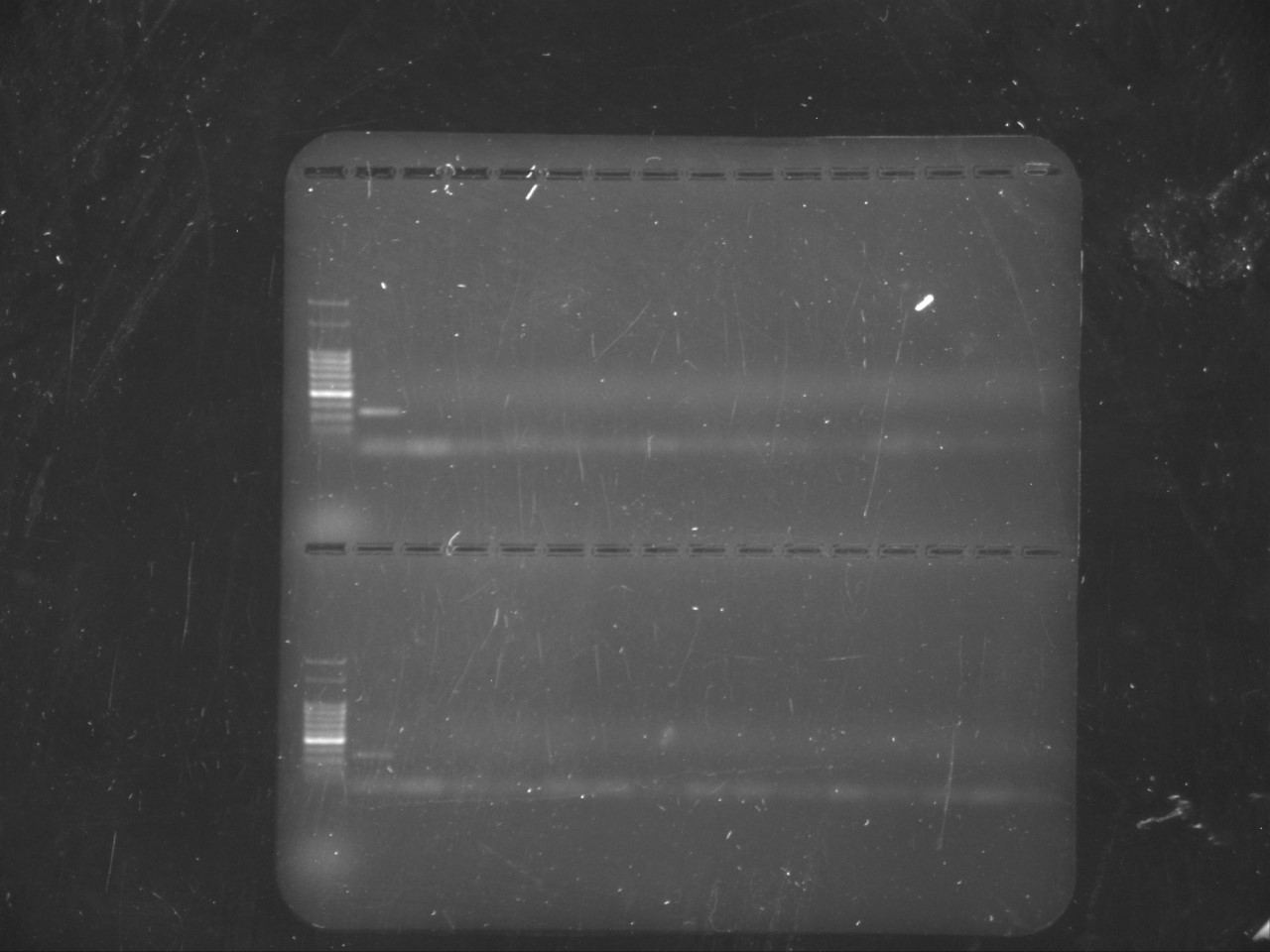
**

**1000-**

**1000-**

**100-**

**500-**

**100-**

**500-**

**(A)**

**(B)**

**Figure 4:** PCR assay for virulence associated genes in enterococci. **(A)** *gelE* (gelatinase enzyme). Lanes 1, 2 and 3: DNA standard, positive control *E. faecalis* ATCC 29212 (419 bp) and negative control, respectively. Lanes 4 to 16: strains 6.9, 6.12, 6.13, 6.14, 6.17, 6.18, 6.19, 6.20, 6.22, 10.1, 10.2, 10.3 and 10.4. Positive strains for amplification are indicated by the asterisks. **(B)** *cylA* (activator of cytolysin). Lanes 1, 2 and 3: DNA standard, positive control *E. faecalis* 8 (517 bp) and negative control, respectively. Lanes 4 to 16: strains 9.21, 9.22, 9.24, 18.1, 18.2, 18.3, 18.4, 18.5, 18.6, 18.7, 18.9, 18.10 and 18.11. **(C)** *esp* (associated to biofilm formation). Lanes 1, 2 and 3: DNA standard, positive control *E. mundtii* 14.3 (1198 bp) and negative control, respectively. Lanes 4 to 16: strains 7.22, 14.18, 11.1, 11.2, 11.3, 11.4, 11.5, 11.6, 11.8, 11.9, 11.10, 11.11, and 11.12. **(D)** *agg* (aggregation substance). Lanes 1, 2 and 3: DNA standard, positive control *E. faecalis* M2.15 (1553 bp) and negative control, respectively. Lanes 4 to 9: strains 7.2, 7.3, 7.4, 7.5 and 7.6. **(E)** *ace* (accessory colonization factor). Lane 1 and 2: positive control *E. faecalis* C12 (320 bp) and negative control, respectively. Lanes 3 to 9: strains 7.3, 7.4, 7.15, 7.19, 6.9, 6.18 and 6.19.

**1 2 3 4 5 6 7 8 9 10 11 12 13 14 15 16**

**STD
bp**

**1500-**

**STD
bp**

**1500-**

**1 2 3 4 5 6 7 8 9 10 11 12 13 14 15 16**


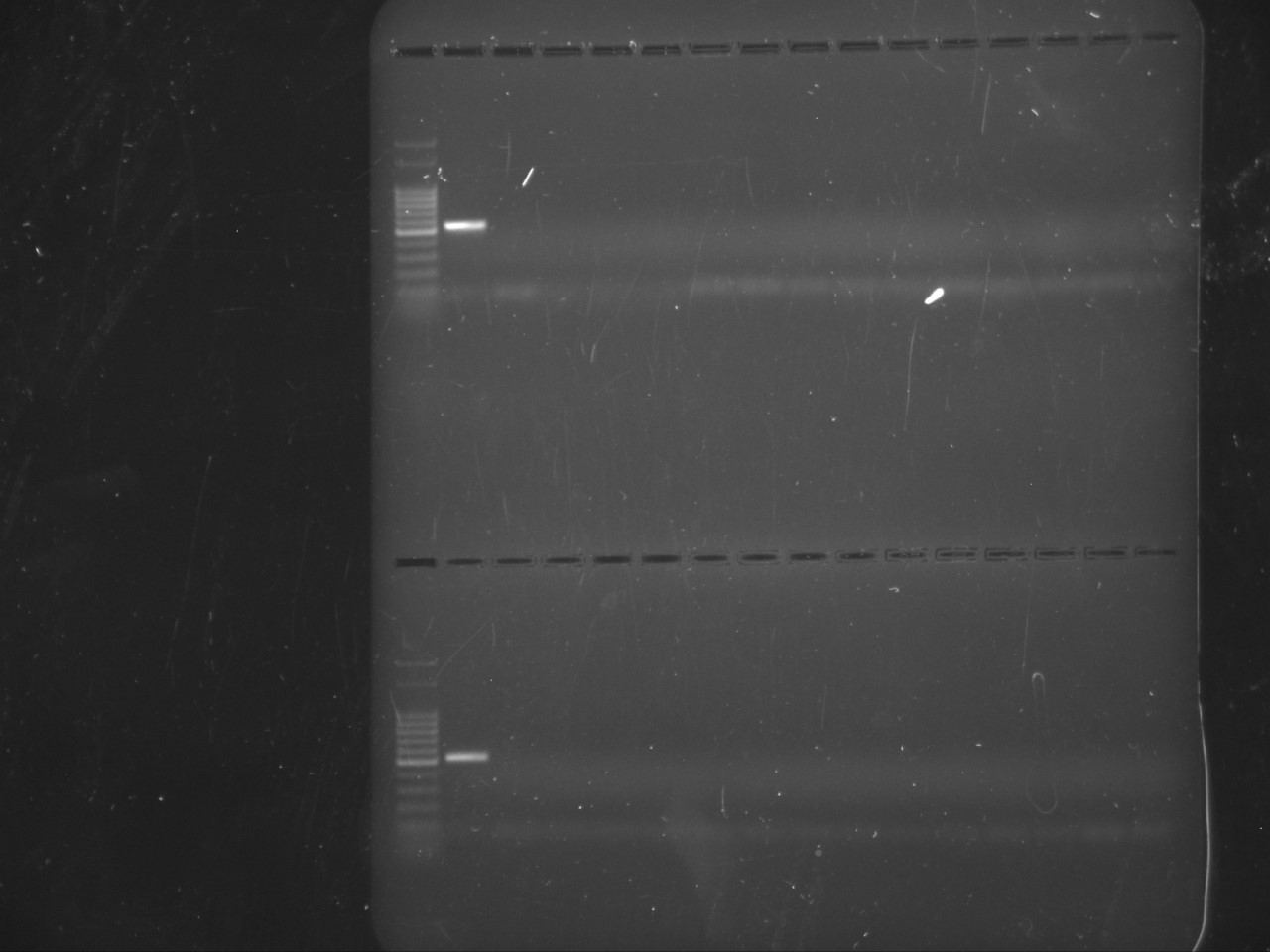

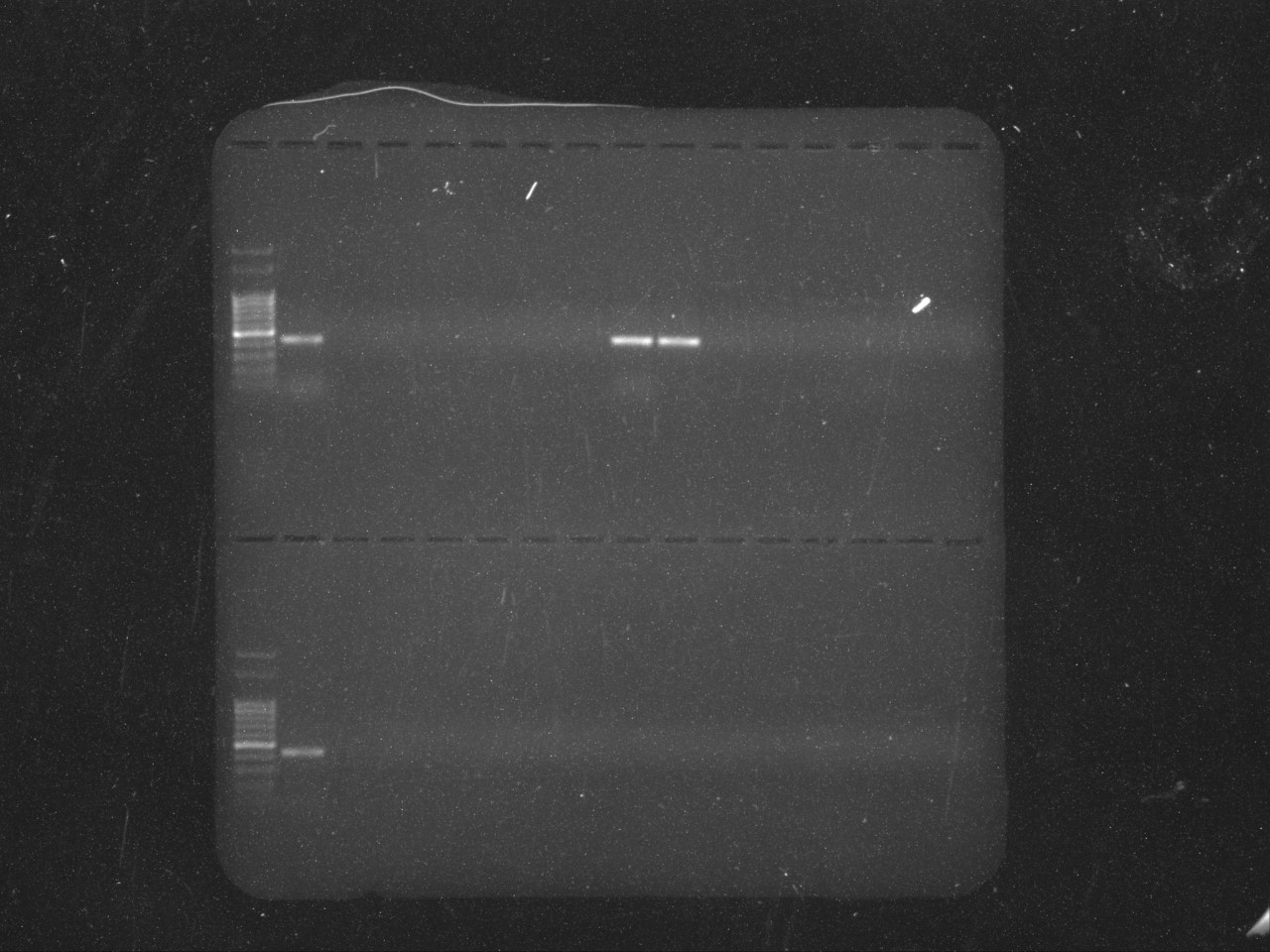


**100-**

**500-**

**1000-**

**100-**

**500-**

**1000-**

**(B)**

**(A)**

*****

*****

**STD
bp**

**1500-**


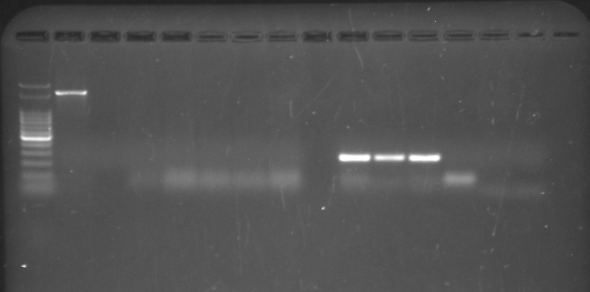

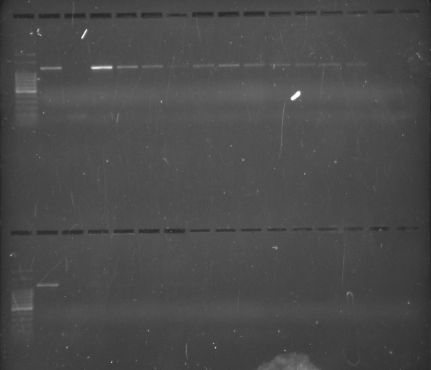


**1 2 3 4 5 6 7 8 9**

**STD
bp**

**1500-**

**1 2 3 4 5 6 7 8 9 10 11 12 13 14 15 16**

**1000-**

**100-**

**500-**

**500-**

**1000-**

**100-**

**(D)**

**(C)**

**1 2 3 4 5 6 7 8 9**


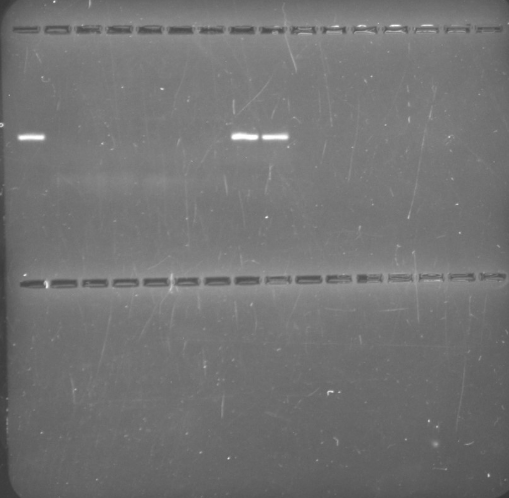


**320 bp-**

**(E)**

**Figure 5:** Cromossomal digestion patterns of enterococci after digestion with *Sma*I. Lane 8: Lambda Ladder PFG Marker (New England Biolabs). Lanes 1 to 15 (except 8): strains 7.21, 7.22, 6.2, 6.4, 10.5, 10.12, 11.9, 7.2, 7.3, 7.19, 7.17, 10.17, 7.16 and 10.8, respectively.

**1 2 3 4 5 6 7 8 9 10 11 12 13 14 15**


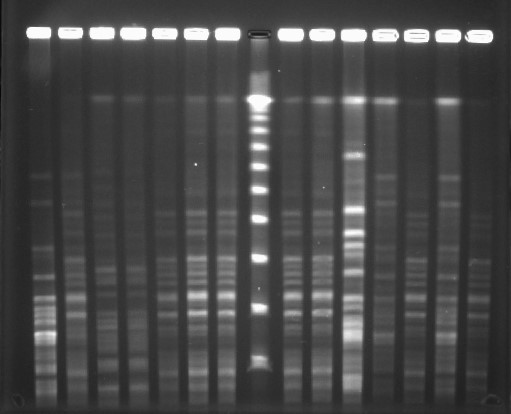

Supplement: Data S1 [file peerj-08-8647-s001.docx]
