# Supplementary material for: Antimicrobial resistance and genetic relationships of enterococci from siblings and non-siblings Heliconius erato phyllis caterpillars
Source: PeerJ. 2020 Feb 27;8:e8647. doi: 10.7717/peerj.8647 (PMC7049460; doi:10.7717/peerj.8647)
Supplement: Data S2 [file peerj-08-8647-s002.docx]

**Raw Data**

**Table 1:**

All enterococci strains isolated from fecal samples of *Heliconius erato phyllis* caterpillars in the present study. Maternal origin, strain identification, enterococci species, antibiotic resistance profile, PCR amplification of genes encoding resistance (*ermC* and *msrC*) and virulence factors (*gelE*, *cylA*, *ace*, *esp* and *agg*), and clonal patterns in PFGE analysis.

| **Origin¹** | **ID** | **Species** | **Antibiotic Resistance Profile²** |  | **Resistance Genes** | |  | **Virulence Genes²** | | | | |  | **PFGE Pattern³** |
| --- | --- | --- | --- | --- | --- | --- | --- | --- | --- | --- | --- | --- | --- | --- |
|  |  |  |  |  | ***erm*(C)** | ***msr*C** |  | ***gel*E** | ***cyl*A** | ***esp*** | ***agg*** | ***ace*** |  |  |
| **HEAB2** | 7.2 | *E. casseliflavus* | NOR, RIF |  | ND | ND |  | - | - | - | - | - |  | P7 |
|  | 7.3 | *E. casseliflavus* | NOR, RIF |  | ND | ND |  | - | - | - | - | - |  | P7 |
|  | 7.4 | *E. casseliflavus* | ERY, RIF |  | - | - |  | - | - | - | - | - |  | ND |
|  | 7.5 | *E. casseliflavus* | RIF |  | ND | ND |  | - | - | - | - | - |  | ND |
|  | 7.6 | *E. casseliflavus* | susceptible |  | ND | ND |  | - | - | - | - | - |  | P12 |
|  | 7.8 | *E. casseliflavus* | susceptible |  | ND | ND |  | - | - | - | - | - |  | ND |
|  | 7.9 | *E. casseliflavus* | susceptible |  | ND | ND |  | - | - | - | - | - |  | P12 |
|  | 7.10 | *E. casseliflavus* | susceptible |  | ND | ND |  | - | - | - | - | - |  | ND |
|  | 7.11 | *E. casseliflavus* | susceptible |  | ND | ND |  | - | - | - | - | - |  | P12 |
|  | 7.12 | *E. casseliflavus* | susceptible |  | ND | ND |  | - | - | - | - | - |  | P8 |
|  | 7.13 | *E. casseliflavus* | ERY, NOR |  | - | - |  | - | - | - | - | - |  | P11 |
|  | 7.15 | *E. casseliflavus* | ERY, RIF |  | - | - |  | - | - | - | - | - |  | P8 |
|  | 7.16 | *E. casseliflavus* | ERY, NOR, CIP |  | - | - |  | - | - | - | - | - |  | P12 |
|  | 7.17 | *E. casseliflavus* | ERY, NOR, RIF |  | - | - |  | - | - | - | - | - |  | P12 |
|  | 7.19 | *E. casseliflavus* | NOR, RIF |  | ND | ND |  | - | - | - | - | - |  | S |
|  | 7.20 | *E. casseliflavus* | ERY |  | - | - |  | - | - | - | - | - |  | P12 |
|  | 7.21 | *E. casseliflavus* | ERY |  | - | - |  | - | - | - | - | - |  | P11 |
|  | 7.22 | *E. casseliflavus* | ERY, RIF |  | - | - |  | - | - | + | - | - |  | S |
|  | 7.23 | *E. casseliflavus* | RIF |  | ND | ND |  | - | - | - | - | - |  | P12 |
|  | 7.24 | *E. casseliflavus* | ERY, RIF |  | - | - |  | - | - | - | - | - |  | ND |
|  | 6.1 | *E. casseliflavus* | ERY, RIF |  | - | - |  | - | - | - | - | - |  | ND |
|  | 6.2 | *E. casseliflavus* | ERY, RIF |  | - | - |  | - | - | - | - | - |  | P5 |
|  | 6.3 | *E. casseliflavus* | ERY, RIF |  | - | - |  | - | - | - | - | - |  | ND |
|  | 6.4 | *E. casseliflavus* | ERY, RIF |  | - | - |  | - | - | - | - | - |  | P5 |
|  | 6.5 | *E. casseliflavus* | ERY, RIF |  | - | - |  | - | - | - | - | - |  | P8 |
|  | 6.8 | *E. casseliflavus* | RIF |  | ND | ND |  | - | - | - | - | - |  | ND |
|  | 6.9 | *E. casseliflavus* | RIF |  | ND | ND |  | - | - | - | - | - |  | ND |
|  | 6.12 | *E. casseliflavus* | RIF |  | ND | ND |  | - | - | - | - | - |  | P8 |
|  | 6.14 | *E. casseliflavus* | RIF |  | ND | ND |  | - | - | - | - | - |  | ND |
|  | 6.17 | *E. casseliflavus* | RIF |  | ND | ND |  | - | - | - | - | - |  | ND |
|  | 6.18 | *E. faecalis* | ERY, RIF |  | - | - |  | + | - | - | - | + |  | P3 |
|  | 6.19 | *E. faecalis* | ERY, RIF |  | - | - |  | + | - | - | - | + |  | P3 |
|  | 6.20 | *E. casseliflavus* | RIF |  | ND | ND |  | - | - | - | - | - |  | P8 |
|  | 6.22 | *E. casseliflavus* | RIF |  | ND | ND |  | - | - | - | - | - |  | ND |
|  | 10.1 | *E. casseliflavus* | RIF |  | ND | ND |  | - | - | + | - | - |  | ND |
|  | 10.2 | *E. casseliflavus* | RIF |  | ND | ND |  | - | - | + | - | - |  | ND |
|  | 10.3 | *E. casseliflavus* | RIF |  | ND | ND |  | - | - | + | - | - |  | ND |
|  | 10.4 | *E. casseliflavus* | ERY, RIF |  | - | - |  | - | - | - | - | - |  | ND |
|  | 10.5 | *E. casseliflavus* | ERY, RIF |  | - | - |  | - | - | + | - | - |  | P7 |
|  | 10.6 | *E. casseliflavus* | RIF |  | ND | ND |  | - | - | - | - | - |  | P8 |
|  | 10.7 | *E. casseliflavus* | ERY, NOR, RIF, CIP |  | - | - |  | - | - | + | - | - |  | P9 |
|  | 10.8 | *E. casseliflavus* | ERY, RIF, CIP |  | - | - |  | - | - | + | - | - |  | P7 |
|  | 10.9 | *E. casseliflavus* | ERY, RIF, CIP |  | - | - |  | - | - | - | - | - |  | S |
|  | 10.10 | *E. casseliflavus* | RIF |  | ND | ND |  | - | - | + | - | - |  | P7 |
|  | 10.11 | *E. casseliflavus* | ERY, RIF |  | - | - |  | - | - | + | - | - |  | ND |
|  | 10.12 | *E. casseliflavus* | ERY, RIF |  | - | - |  | - | - | + | - | - |  | P7 |
|  | 10.13 | *E. casseliflavus* | ERY, RIF |  | - | - |  | - | - | - | - | - |  | ND |
|  | 10.14 | *E. casseliflavus* | RIF |  | ND | ND |  | - | - | - | - | - |  | ND |
|  | 10.15 | *E. casseliflavus* | RIF |  | ND | ND |  | - | - | - | - | - |  | ND |
|  | 10.16 | *E. casseliflavus* | ERY, RIF |  | - | - |  | - | - | + | - | - |  | ND |
|  | 10.17 | *E. casseliflavus* | ERY, NOR, RIF |  | - | - |  | - | - | - | - | - |  | P7 |
|  | 10.18 | *E. casseliflavus* | RIF |  | ND | ND |  | - | - | + | - | - |  | ND |
|  | 10.19 | *E. casseliflavus* | RIF |  | ND | ND |  | - | - | + | - | - |  | ND |
|  | 10.20 | *E. casseliflavus* | RIF |  | ND | ND |  | - | - | + | - | - |  | ND |
|  | 10.30 | *E. casseliflavus* | RIF |  | ND | ND |  | - | - | + | - | - |  | ND |
|  | 10.31 | *E. casseliflavus* | RIF |  | ND | ND |  | - | - | + | - | - |  | ND |
|  | 10.32 | *E. casseliflavus* | RIF |  | ND | ND |  | - | - | + | - | - |  | ND |
|  | 10.33 | *E. casseliflavus* | RIF |  | ND | ND |  | - | - | + | - | - |  | ND |
|  | 10.34 | *E. casseliflavus* | RIF |  | ND | ND |  | - | - | + | - | - |  | ND |
|  | 10.35 | *E. casseliflavus* | RIF |  | ND | ND |  | - | - | + | - | - |  | ND |
|  | 11.1 | *E. casseliflavus* | RIF |  | ND | ND |  | - | - | + | - | - |  | ND |
|  | 11.2 | *E. casseliflavus* | RIF |  | ND | ND |  | - | - | + | - | - |  | ND |
|  | 11.3 | *E. casseliflavus* | RIF |  | ND | ND |  | - | - | + | - | - |  | ND |
|  | 11.4 | *E. casseliflavus* | RIF |  | ND | ND |  | - | - | + | - | - |  | ND |
|  | 11.5 | *E. casseliflavus* | RIF |  | ND | ND |  | - | - | + | - | - |  | ND |
|  | 11.6 | *E. casseliflavus* | susceptible |  | ND | ND |  | - | - | + | - | - |  | P9 |
|  | 11.8 | *E. casseliflavus* | RIF |  | ND | ND |  | - | - | + | - | - |  | P7 |
|  | 11.9 | *E. casseliflavus* | ERY, RIF |  | - | - |  | - | - | + | - | - |  | P7 |
|  | 11.10 | *E. casseliflavus* | RIF |  | ND | ND |  | - | - | + | - | - |  | ND |
|  | 11.11 | *E. casseliflavus* | RIF |  | ND | ND |  | - | - | - | - | - |  | ND |
|  | 11.12 | *E. casseliflavus* | RIF |  | ND | ND |  | - | - | - | - | - |  | ND |
|  | 11.13 | *E. casseliflavus* | RIF |  | ND | ND |  | - | - | - | - | - |  | ND |
|  | 11.14 | *E. casseliflavus* | RIF |  | ND | ND |  | - | - | - | - | - |  | ND |
|  | 11.15 | *E. casseliflavus* | RIF |  | ND | ND |  | - | - | - | - | - |  | ND |
|  | 11.16 | *E. casseliflavus* | RIF |  | ND | ND |  | - | - | - | - | - |  | ND |
|  | 11.17 | *E. casseliflavus* | RIF |  | ND | ND |  | - | - | - | - | - |  | ND |
|  | 11.18 | *E. casseliflavus* | RIF |  | ND | ND |  | - | - | - | - | - |  | P9 |
|  | 11.19 | *E. casseliflavus* | RIF |  | ND | ND |  | - | - | - | - | - |  | ND |
|  | 11.20 | *E. casseliflavus* | RIF |  | ND | ND |  | - | - | - | - | - |  | ND |
|  | 11.21 | *E. casseliflavus* | RIF |  | ND | ND |  | - | - | - | - | - |  | ND |
|  | 11.22 | *E. casseliflavus* | RIF |  | ND | ND |  | - | - | - | - | - |  | ND |
|  | 11.23 | *E. casseliflavus* | RIF |  | ND | ND |  | - | - | - | - | - |  | ND |
|  | 11.24 | *E. casseliflavus* | RIF |  | ND | ND |  | - | - | - | - | - |  | ND |
|  | 14.1 | *E. mundtii* | susceptible |  | ND | ND |  | - | - | + | - | - |  | P10 |
|  | 14.2 | *E. mundtii* | susceptible |  | ND | ND |  | - | - | + | - | - |  | ND |
|  | 14.3 | *E. mundtii* | susceptible |  | ND | ND |  | - | - | + | - | - |  | P10 |
|  | 14.4 | *E. mundtii* | susceptible |  | ND | ND |  | - | - | + | - | - |  | ND |
|  | 14.5 | *E. mundtii* | susceptible |  | ND | ND |  | - | - | + | - | - |  | P10 |
|  | 14.7 | *E. mundtii* | susceptible |  | ND | ND |  | - | - | + | - | - |  | P10 |
|  | 14.8 | *E. mundtii* | susceptible |  | ND | ND |  | - | - | + | - | - |  | ND |
|  | 14.9 | *E. mundtii* | susceptible |  | ND | ND |  | - | - | + | - | - |  | P10 |
|  | 14.10 | *E. mundtii* | susceptible |  | ND | ND |  | - | - | + | - | - |  | ND |
|  | 14.12 | *E. mundtii* | susceptible |  | ND | ND |  | - | - | - | - | - |  | P10 |
|  | 14.14 | *E. mundtii* | susceptible |  | ND | ND |  | - | - | + | - | - |  | P10 |
|  | 14.15 | *E. casseliflavus* | susceptible |  | ND | ND |  | - | - | + | - | - |  | ND |
|  | 14.16 | *E. mundtii* | susceptible |  | ND | ND |  | - | - | + | - | - |  | P10 |
|  | 14.17 | *E. mundtii* | susceptible |  | ND | ND |  | - | - | + | - | - |  | ND |
|  | 14.18 | *E. casseliflavus* | susceptible |  | ND | ND |  | - | - | + | - | - |  | ND |
| **HEV2** | 9.1 | *E. casseliflavus* | susceptible |  | ND | ND |  | - | - | - | - | - |  | P15 |
|  | 9.2 | *E. casseliflavus* | ERY |  | - | - |  | - | - | + | - | - |  | ND |
|  | 9.3 | *E. casseliflavus* | susceptible |  | ND | ND |  | - | - | + | - | - |  | P15 |
|  | 9.4 | *E. casseliflavus* | susceptible |  | ND | ND |  | - | - | - | - | - |  | P15 |
|  | 9.5 | *E. casseliflavus* | ERY |  | - | - |  | - | - | + | - | - |  | P15 |
|  | 9.6 | *E. casseliflavus* | ERY |  | - | - |  | - | - | + | - | - |  | ND |
|  | 9.7 | *E. casseliflavus* | ERY |  | - | - |  | - | - | - | - | - |  | ND |
|  | 9.8 | *E. casseliflavus* | ERY |  | - | - |  | - | - | + | - | - |  | ND |
|  | 9.9 | *E. casseliflavus* | ERY |  | - | - |  | - | - | - | - | - |  | P15 |
|  | 9.10 | *E. casseliflavus* | ERY |  | - | - |  | - | - | - | - | - |  | ND |
|  | 9.14 | *E. casseliflavus* | ERY |  | - | - |  | - | - | - | - | - |  | P15 |
|  | 9.15 | *E. casseliflavus* | ERY |  | - | - |  | - | - | - | - | - |  | ND |
|  | 9.17 | *E. casseliflavus* | ERY |  | - | - |  | - | - | - | - | - |  | P15 |
|  | 9.18 | *E. casseliflavus* | ERY |  | - | - |  | - | - | - | - | - |  | ND |
|  | 9.19 | *E. casseliflavus* | ERY |  | - | - |  | - | - | - | - | - |  | ND |
|  | 9.20 | *E. casseliflavus* | ERY |  | - | - |  | - | - | - | - | - |  | P15 |
|  | 9.21 | *E. casseliflavus* | ERY |  | - | - |  | - | - | - | - | - |  | ND |
|  | 9.22 | *E. casseliflavus* | ERY |  | - | - |  | - | - | - | - | - |  | ND |
|  | 9.24 | *E. casseliflavus* | ERY |  | - | - |  | - | - | - | - | - |  | P15 |
|  | 18.1 | *E. casseliflavus* | ERY, RIF |  | - | - |  | - | - | - | - | - |  | ND |
|  | 18.2 | *E. casseliflavus* | ERY, RIF |  | - | - |  | - | - | - | - | - |  | S |
|  | 18.3 | *Enterococcus* sp. | RIF |  | ND | ND |  | - | - | - | - | - |  | P13 |
|  | 18.4 | *E. casseliflavus* | ERY, RIF |  | - | - |  | - | - | - | - | - |  | P4 |
|  | 18.5 | *E. casseliflavus* | RIF |  | ND | ND |  | - | - | - | - | - |  | ND |
|  | 18.6 | *E. casseliflavus* | ERY, RIF |  | - | - |  | - | - | - | - | - |  | P4 |
|  | 18.7 | *E. casseliflavus* | ERY, RIF |  | - | - |  | - | - | - | - | - |  | ND |
|  | 18.9 | *E. casseliflavus* | RIF |  | ND | ND |  | - | - | - | - | - |  | ND |
|  | 18.10 | *Enterococcus* sp. | RIF |  | ND | ND |  | - | - | - | - | - |  | S |
|  | 18.11 | *E. casseliflavus* | RIF |  | ND | ND |  | - | - | - | - | - |  | ND |
|  | 18.13 | *E. casseliflavus* | RIF |  | ND | ND |  | - | - | - | - | - |  | ND |
|  | 18.14 | *E. casseliflavus* | RIF |  | ND | ND |  | - | - | - | - | - |  | ND |
|  | 18.15 | *E. casseliflavus* | RIF |  | ND | ND |  | - | - | - | - | - |  | P4 |
|  | 18.16 | *E. casseliflavus* | ERY, RIF |  | - | - |  | - | - | - | - | - |  | P4 |
|  | 18.17 | *E. casseliflavus* | ERY, RIF |  | - | - |  | - | - | - | - | - |  | ND |
|  | 18.18 | *E. casseliflavus* | ERY, RIF |  | - | - |  | - | - | - | - | - |  | P4 |
|  | 18.19 | *E. casseliflavus* | ERY, RIF |  | - | - |  | - | - | - | - | - |  | ND |
|  | 18.20 | *E. casseliflavus* | ERY, RIF |  | - | - |  | - | - | - | - | - |  | ND |
|  | 18.21 | *E. casseliflavus* | ERY, RIF |  | - | - |  | - | - | - | - | - |  | P4 |
|  | 26.1 | *E. mundtii* | susceptible |  | ND | ND |  | - | - | + | - | - |  | ND |
|  | 26.2 | *E. casseliflavus* | RIF |  | ND | ND |  | - | - | - | - | - |  | P6 |
|  | 26.3 | *E. casseliflavus* | RIF |  | ND | ND |  | - | - | - | - | - |  | ND |
|  | 26.5 | *Enterococcus* sp. | RIF |  | ND | ND |  | - | - | - | - | - |  | S |
|  | 26.6 | *E. mundtii* | susceptible |  | ND | ND |  | - | - | - | - | - |  | ND |
|  | 26.8 | *E. casseliflavus* | RIF |  | ND | ND |  | - | - | - | - | - |  | ND |
|  | 26.9 | *E. casseliflavus* | RIF |  | ND | ND |  | - | - | + | - | - |  | P6 |
|  | 26.11 | *E. mundtii* | susceptible |  | ND | ND |  | - | - | + | - | - |  | ND |
|  | 26.12 | *E. mundtii* | susceptible |  | ND | ND |  | - | - | + | - | - |  | ND |
|  | 26.14 | *E. mundtii* | susceptible |  | ND | ND |  | - | - | + | - | - |  | P13 |
|  | 26.15 | *E. mundtii* | susceptible |  | ND | ND |  | - | - | + | - | - |  | ND |
|  | 26.18 | *E. casseliflavus* | RIF |  | ND | ND |  | - | - | - | - | - |  | ND |
|  | 26.19 | *E. mundtii* | susceptible |  | ND | ND |  | - | - | + | - | - |  | P13 |
|  | 26.20 | *E. casseliflavus* | RIF |  | ND | ND |  | - | - | - | - | - |  | P6 |
|  | 26.21 | *E. mundtii* | susceptible |  | ND | ND |  | - | - | - | - | - |  | P13 |
|  | 27.3 | *E. mundtii* | susceptible |  | ND | ND |  | - | - | - | - | + |  | P13 |
|  | 27.4 | *E. mundtii* | susceptible |  | ND | ND |  | - | - | - | - | - |  | ND |
|  | 27.8 | *E. mundtii* | susceptible |  | ND | ND |  | - | - | + | - | + |  | ND |
|  | 27.16 | *E. mundtii* | susceptible |  | ND | ND |  | - | - | - | - | + |  | P13 |
|  | 27.17 | *E. mundtii* | susceptible |  | ND | ND |  | - | - | - | - | - |  | ND |
|  | 27.18 | *E. mundtii* | susceptible |  | ND | ND |  | - | - | + | - | - |  | ND |
|  | 29.2 | *E. mundtii* | susceptible |  | ND | ND |  | - | - | + | - | - |  | P13 |
|  | 29.10 | *E. mundtii* | susceptible |  | ND | ND |  | - | - | - | - | + |  | ND |
|  | 29.14 | *E. mundtii* | susceptible |  | ND | ND |  | - | - | - | - | - |  | P13 |
|  | 29.19 | *E. mundtii* | susceptible |  | ND | ND |  | - | - | - | - | + |  | P13 |
|  | 29.20 | *E. mundtii* | susceptible |  | ND | ND |  | - | - | - | - | + |  | P13 |
| **HES2** | 3.2 | *E. casseliflavus* | susceptible |  | ND | ND |  | - | - | - | - | - |  | P1 |
|  | 3.4 | *E. mundtii* | susceptible |  | ND | ND |  | - | - | - | - | - |  | P14 |
|  | 3.15 | *E. casseliflavus* | susceptible |  | ND | ND |  | - | - | - | - | - |  | P1 |
|  | 3.16 | *E. mundtii* | susceptible |  | ND | ND |  | - | - | + | - | - |  | P14 |
|  | 3.18 | *E. mundtii* | susceptible |  | ND | ND |  | - | - | + | - | + |  | P14 |
|  | 3.19 | *E. mundtii* | susceptible |  | ND | ND |  | - | - | + | - | + |  | P14 |
|  | 3.20 | *E. casseliflavus* | susceptible |  | ND | ND |  | - | - | - | - | - |  | P1 |
|  | 3.21 | *E. mundtii* | susceptible |  | ND | ND |  | - | - | + | - | + |  | P14 |
|  | 3.22 | *E. casseliflavus* | susceptible |  | ND | ND |  | - | - | - | - | - |  | P1 |
|  | 3.24 | *E. mundtii* | susceptible |  | ND | ND |  | - | - | + | - | + |  | S |
|  | 17.10 | *E. casseliflavus* | susceptible |  | ND | ND |  | - | - | - | - | - |  | P2 |
|  | 17.14 | *E. casseliflavus* | susceptible |  | ND | ND |  | - | - | - | - | - |  | P2 |
|  | 17.16 | *E. casseliflavus* | susceptible |  | ND | ND |  | - | - | - | - | - |  | P2 |
|  | 17.18 | *Enterococcus* sp. | ERY, RIF, CIP |  | - | - |  | - | - | - | - | - |  | ND |
|  | 17.20 | *Enterococcus* sp. | susceptible |  | ND | ND |  | - | - | - | - | - |  | ND |
|  | 17.23 | *Enterococcus* sp. | RIF |  | ND | ND |  | - | - | - | - | - |  | ND |

¹Origin: HEAB2, female from Águas Belas; HEV2*,* female from Viamão; HES2*,* female from São Francisco de Paula. ²Antibiotic Resistance Profile: ERY, erythromycin; CIP, ciprofloxacin; NOR, norfloxacin; RIF, rifampicin; susceptible, strain susceptible to all antibiotic tested. ³PFGE Pattern: S, singleton; P: clonal profile; ND: not determined. + : positive amplification; - : negative amplification.
