# Supplementary material for: Antimicrobial resistance and genetic relationships of enterococci from siblings and non-siblings Heliconius erato phyllis caterpillars
Source: PeerJ. 2020 Feb 27;8:e8647. doi: 10.7717/peerj.8647 (PMC7049460; doi:10.7717/peerj.8647)
Supplement: Table S1 [file peerj-08-8647-s003.docx]

**Supplemental Information**

**Table S1:**

Origin, identification and antimicrobial profile of enterococci strains chosen for PFGE analysis in this study.

| **Female¹** | **Caterpillar** | **Oviposition date** | **Species** | **Identification** | **Antimicrobial profile²** | | | | | | | **PFGE patterns** |
| --- | --- | --- | --- | --- | --- | --- | --- | --- | --- | --- | --- | --- |
|  |  |  |  |  | **RIF** | **ERY** | | | **NOR** | | **CIP** |  |
| **HEAB2** | 6 | 11/20/2014 | *E. casseliflavus* | 6.2; 6.4 | R | | R | S | | S | | P5 |
|  |  |  |  | 6.5 | R | | R | S | | S | | P8 |
|  |  |  |  | 6.12; 6.20 | R | | S | S | | S | | P8 |
|  |  |  | *E. faecalis* | 6.18; 6.19 | R | | R | S | | S | | P3 |
|  | 7 | 11/21/2014 | *E. casseliflavus* | 7.2; 7.3 | R | | S | R | | S | | P7 |
|  |  |  |  | 7.19 | R | | S | R | | S | | singleton |
|  |  |  |  | 7.6; 7.9; 7.11 | S | | S | S | | S | | P12 |
|  |  |  |  | 7.13 | S | | R | R | | S | | P11 |
|  |  |  |  | 7.16 | S | | R | R | | R | | P12 |
|  |  |  |  | 7.17 | R | | R | R | | S | | P12 |
|  |  |  |  | 7.20 | S | | R | S | | S | | P12 |
|  |  |  |  | 7.21 | S | | R | S | | S | | P11 |
|  |  |  |  | 7.22 | R | | R | S | | S | | singleton |
|  |  |  |  | 7.23 | R | | S | S | | S | | P12 |
|  |  |  |  | 7.12 | S | | S | S | | S | | P8 |
|  |  |  |  | 7.15 | R | | R | S | | S | | P8 |
|  | 10 | 11/22/2014 | *E. casseliflavus* | 10.6 | R | | S | S | | S | | P8 |
|  |  |  |  | 10.5; 10.12 | R | | R | S | | S | | P7 |
|  |  |  |  | 10.7 | R | | R | R | | R | | P9 |
|  |  |  |  | 10.8 | R | | R | S | | R | | P7 |
|  |  |  |  | 10.9 | R | | R | S | | R | | singleton |
|  |  |  |  | 10.10 | R | | S | S | | S | | P7 |
|  |  |  |  | 10.17 | R | | R | R | | S | | P7 |
|  | 11 | 11/23/2014 | *E. casseliflavus* | 11.6 | S | | S | S | | S | | P9 |
|  |  |  |  | 11.8 | R | | S | S | | S | | P7 |
|  |  |  |  | 11.9 | R | | R | S | | S | | P7 |
|  |  |  |  | 11.18 | R | | S | S | | S | | P9 |
|  | 14 | 11/28/2014 | *E. mundtii* | 14.1; 14.3; 14.5; 14.7; 14.9; 14.12; 14.14; 14.16 | S | | S | S | | S | | P10 |
| **HEV2** | 9 | 02/04/2015 | *E. casseliflavus* | 9.1; 9.3; 9.4 | S | | S | S | | S | | P15 |
|  |  |  |  | 9.5; 9.9; 9.14; 9.17; 9.20; 9.24 | S | | R | S | | S | | P15 |
|  | 18 | 02/12/2015 | *E. casseliflavus* | 18.2 | R | | R | S | | S | | singleton |
|  |  |  |  | 18.4; 18.6; 18.16; 18.18; 18.21 | R | | R | S | | S | | P4 |
|  |  |  |  | 18.15 | R | | S | S | | S | | P4 |
|  |  |  | *Enterococcus* sp. | 18.3 | R | | S | S | | S | | P13 |
|  |  |  |  | 18.10 | R | | S | S | | S | | singleton |
|  | 26 | 02/28/2015 | *E. casseliflavus* | 26.2; 26.9; 26.20 | R | | S | S | | S | | P6 |
|  |  |  | *Enterococcus* sp. | 26.5 | R | | S | S | | S | | singleton |
|  |  |  | *E. mundtii* | 26.14; 26.19; 26.21 | S | | S | S | | S | | P13 |
|  | 27 | 02/26/2015 | *E. mundtii* | 27.3; 27.16 | S | | S | S | | S | | P13 |
|  | 29 | 03/02/2015 | *E. mundtii* | 29.2; 29.14; 29.19; 29.20 | S | | S | S | | S | | P13 |
| **HES2** | 3 | * | *E. casseliflavus* | 3.2; 3.15; 3.20; 3.22 | S | | S | S | | S | | P1 |
|  |  |  | *E. mundtii* | 3.4; 3.16; 3.18; 3.19; 3.21 | S | | S | S | | S | | P14 |
|  |  |  |  | 3.24 | S | | S | S | | S | | singleton |
|  | 17 | 04/01/2015 | *E. casseliflavus* | 17.10; 17.14; 17.16 | S | | S | S | | S | | P2 |

¹HEAB2, Female from Águas Belas; HEV2*,* female from Viamão; HES2*,* female from São Francisco de Paula. ²RIF, Rifampicin (5 μg); ERY, Erythromycin (15 μg); NOR, Norfloxacin (10 μg); CIP, Ciprofloxacin (5 μg). S, Susceptible; R, Resistant. All strains were susceptible to gentamicin (120 μg), tetracycline (30 μg), streptomycin (300 μg), vancomycin (30 μg), chloramphenicol (30 μg), nitrofurantoin (300 μg) and ampicillin (10 μg). *data not available.
